# Analysis of Gal4 Expression Patterns in Adult *Drosophila* Females

**DOI:** 10.1534/g3.120.401676

**Published:** 2020-09-11

**Authors:** Lesley N. Weaver, Tianlu Ma, Daniela Drummond-Barbosa

**Affiliations:** Department of Biochemistry and Molecular Biology, Bloomberg School of Public Health, Johns Hopkins University, Baltimore, MD 21205

**Keywords:** Gal4, *Drosophila*, tissue-specific expression, adult, female

## Abstract

Precise genetic manipulation of specific cell types or tissues to pinpoint gene function requirement is a critical step in studies aimed at unraveling the intricacies of organismal physiology. *Drosophila* researchers heavily rely on the *UAS/Gal4/Gal80* system for tissue-specific manipulations; however, it is often unclear whether the reported Gal4 expression patterns are indeed specific to the tissue of interest such that experimental results are not confounded by secondary sites of Gal4 expression. Here, we surveyed the expression patterns of commonly used *Gal4* drivers in adult *Drosophila* female tissues under optimal conditions and found that multiple drivers have unreported secondary sites of expression beyond their published cell type/tissue expression pattern. These results underscore the importance of thoroughly characterizing Gal4 tools as part of a rigorous experimental design that avoids potential misinterpretation of results as we strive for understanding how the function of a specific gene/pathway in one tissue contributes to whole-body physiology.

Organismal physiology involves extensive inter-organ communication via circulating factors that are produced and secreted in response to changes in the local, systemic, or external environment. Many organs can sense and communicate such changes by sending signals to other tissues to ensure whole-body homeostasis (Droujinine and Perrimon 2016). For example, growth-blocking peptides produced in the larval fat body (in response to dietary amino acids) activate the epidermal growth factor receptor in inhibitory neurons connected to insulin-producing cells to facilitate insulin secretion (Meschi *et al.* 2019). Activin-β secreted from enteroendocrine cells in the midgut (in response to a high sugar diet) enhances the response of the fat body to adipokinetic hormone (the *Drosophila* glucagon analog), resulting in hyperglycemia in larvae (Song *et al.* 2017). In adults, it was recently shown that ecdysone produced in the ovary stimulates intestinal stem cell (ISC) division in mated females (Ahmed *et al.* 2020). Oogenesis itself is highly sensitive to changes in physiology and can be modulated by manipulations in peripheral tissues, including the fat body (Armstrong *et al.* 2014; Matsuoka *et al.* 2017; Armstrong and Drummond-Barbosa 2018; Weaver and Drummond-Barbosa 2018; Weaver and Drummond-Barbosa 2019), gut (Ameku *et al.* 2018), and brain (Lafever and Drummond-Barbosa 2005; Sieber and Spradling 2015). Studies aimed at understanding the complex endocrine relationships among organs as organisms respond to physiological or environmental changes require experimental tools that allow cell type/tissue-specific manipulations.

The *UAS/Gal4/Gal80* system is commonly used in *Drosophila* to manipulate a specific cell type or tissue to determine the requirements for genes and pathways either in regulating that same cell type/tissue of interest or in remotely affecting separate tissues (Brand and Perrimon 1993). The *UAS/Gal4/Gal80* system employs the yeast transcription factor *Gal4* under the control of a “tissue-specific” enhancer/promoter sequence (referred to as the “driver”) in combination with a “responder” that contains an *Upstream Activating Sequence* composed of Gal4 binding sites upstream of a target gene or sequence of interest (Brand and Perrimon 1993). Gal4 binds to the *UAS* sequence, thereby inducing tissue-specific expression of the transgene (*e.g.*, fluorescent reporter, hairpin RNA, protein-coding gene, etc). The Gal4 inhibitor *Gal80* (Douglas and Hawthorne 1966) can be added to this system for multiple purposes. For example, expression of Gal80 under a tissue-specific promoter can be used to inhibit Gal4 function in a subset of cell types/tissues to generate a more tissue/cell type specific driver (Eliason *et al.* 2018). Loss of a *Gal80* transgene can also be used for the generation of Flp/FRT-induced positively marked loss-of-function clones (expressing a fluorescent reporter driven by Gal4) during genetic mosaic analysis (Lee and Luo 1999). In addition, a temperature-sensitive *Gal80* mutant allele can be used to temporally restrict Gal4 activity to specific developmental stages (*e.g.*, larvae or adults) or experimental time windows (McGuire *et al.* 2003). This system has been instrumental in the use of *Drosophila* as a model for understanding complex cellular and physiological processes.

A potential caveat to the *Gal4/UAS* system, however, is that the described cell type- or tissue-specific Gal4 expression patterns can be incomplete, such that published Gal4 lines might have additional unreported sites of expression that could potentially confound the interpretation of experimental results. In fact, when previously assessing published fat body-specific drivers in adult females to identify an adipocyte-specific Gal4, we found that the majority of those drivers were expressed in additional tissues besides the fat body in adult females (Armstrong *et al.* 2014). As this example illustrates, scientists studying adult female physiology would benefit from having a set of commonly used *Gal4* drivers that have been thoroughly analyzed for their expression patterns in adult females, such that their tissue specificity is unequivocal.

In this study, we selected commonly used *Gal4* drivers and analyzed their expression patterns in all of the major tissues of the adult *Drosophila* female. We found that a significant number of *Gal4* drivers typically used for the genetic manipulation of specific cell types in the ovary or midgut have previously unreported expression in additional, secondary tissues. By contrast, most of the *Gal4* drivers for neuronal subpopulations are indeed specific, as they show their reported pattern without expression in additional tissues. Finally, we highlight techniques commonly used in *Drosophila* for inhibiting Gal4 expression in secondary tissues, as well as other ways to rule out secondary tissue effects when Gal4 is expressed in multiple tissues.

## Materials And Methods

### Drosophila strains and culture conditions

*Drosophila* stocks were maintained at room temperature (22-25°) on standard medium containing cornmeal, molasses, yeast, and agar. Previously described *Gal4* lines used in this study are included in Table 1. The *nSyb-Gal80* transgene has been previously described (Rubinstein *et al.* 2010). The *UAS-GFP.nls* (*w^1118^*; *P{UAS-GFP.nls}14*), *UAS-mCD8*::*GFP* (w*; *P{10XUAS-IVS-mCD8*::*GFP}attP2*), and *UASp-lacZ* lines were obtained from the Bloomington *Drosophila* Stock Center (BDSC; bdsc.indiana.edu/). Additional genetic elements are described in FlyBase (http://www.flybase.org).

**Table 1:** Full genotypes of *Gal4* drivers used in this study

Driver	Genotype	Source	Reference
*bab1-Gal4*	*w**^a^*; *P{w^+mW.hs^ = GawB}bab1^Agal4-5^]/TM3*, *Sb^1^*	BDSC 6802	(Cabrera *et al.* 2002)
*hh-Gal4^MB^*	*sp/CyO*; *hh-Gal4/TM3*	Michael Buszczak	(Eliazer *et al.* 2011)
*hh-Gal4^TX^*	*w*; *hh-Gal4/TM6B*	Ting Xie	(Pan *et al.* 2007)
*hh-Gal4^JF^*	*w^1118^*; *P{y^+t7.7^ w^+mC^ = GMR28E03-GAL4}attP2*	BDSC 45546	(Jenett *et al.* 2012)
*ptc-Gal4*	*ptc-Gal4/CyO act-GFP*; *tub-Gal80^ts^/TM6B*	D.D.-B. Lab*^a^*	(Forbes *et al.* 1996)
*c587-Gal4*	*c587-Gal4/FM7i*; *tub-Gal80^ts^/CyO*, *Act-GFP*	D.D.-B. Lab	(Hsu and Drummond-Barbosa 2009)
*tj-Gal4*	*tj-Gal4 tub-Gal80^ts^/CyO twist-gal4.UAS-GFP*	D.D.-B. Lab	(Sahai-Hernandez and Nystul 2013)
*mex1-Gal4*	*mex-Gal4/TM6B*	Allan Spradling	(Phillips and Thomas 2006)
*NP3084-Gal4*	*w**^a^*; *P{GawB}NP3084*	Kyoto 113094	(Hayashi *et al.* 2002)
*esg-Gal4*	*esg-Gal4*; *tub-Gal80^ts^ UAS-GFP*	Allan Spradling	(Micchelli and Perrimon 2006)
*dl-Gal4*	*y w*; *tub-Gal80^ts^/CyO*; *delta-Gal4/TM3*	Benoit Biteau	(Zeng *et al.* 2010)
*Su(H)GBE-Gal4*	*y w*; *GBE Su(H)-Gal4 UAS-GFP/CyO*; *tub-Gal80^ts^/TM3*	Benoit Biteau	(Zeng *et al.* 2010)
*c42-Gal4*	*w**^a^*; *P{w^+mW.hs^ = GawB}c42*	BDSC 30835	(Rosay *et al.* 1997)
*Uro-Gal4*	*w**^a^*; *P{Uro-GAL4.T}2*	BDSC 44416	(Terhzaz *et al.* 2010)
*mef2-Gal4*	*tub-Gal80^ts^/CyO*; *mef2-Gal4/TM6B*	D.D.-B. Lab	(Ranganayakulu *et al.* 1998)
*nSyb.P-Gal4*	*y^1^ w^1118^*; *P{y^+t7.7^ w^+mC^ = nSyb-GAL4.P}attP2*	BDSC 51941	(Riabinina *et al.* 2015)
*nSyb.S-Gal4*	*y^1^ w**^a^*; *P{w^+m^**^a^* *=nSyb-GAL4.S}3*	Mark Wu	(Liu *et al.* 2012)
*repo-Gal4*	*w^1118^*; *P{w^+m^**^a^* *=GAL4}repo/TM3*, *Sb^1^*	BDSC 7415	(Sepp *et al.* 2001)
*ChAT-Gal4*	*w^1118^*; *P{w^+mC^ = ChAT-GAL4.7.4}19B/CyO*, *P{ry^+t7.2^ = sevRas1.V12}FK1*	BDSC 6798	(Salvaterra and Kitamoto 2001)
*pebbled-Gal4*	*w**^a^* *P{w^+m^**^a^**=GAL4}peb*	Chris Potter	(Sweeney *et al.* 2007)
*Gr5a-Gal4*	*pin/CyO*; *Gr5a-Gal4/TM6b*	Chris Potter	(Wang *et al.* 2004)
*Gr66a-Gal4*	*w*^-^; *Gr66a-Gal4*; *GR93a^3^*	Chris Potter	(Wang *et al.* 2004)
*Ir8a-Gal4*	*Ir8a-Gal4/CyO*	Chris Potter	(Abuin *et al.* 2011)
*Ir25a-Gal4*	*Ir25a-Gal4/CyO*	Chris Potter	(Abuin *et al.* 2011)
*Or83b-Gal4*	*w*^-^; *Or83b-Gal4/CyO*	Chris Potter	(Wang *et al.* 2004)
*ppk23-Gal4*	*Bl/CyO*; *ppk23-Gal4/TM6b*	Chris Potter	(Wang *et al.* 2004)
*tub-Gal4*	*y w*; *tub-Gal80^ts^*; *tub-Gal4/TM6B*	D.D.-B. lab	(Nabel-Rosen *et al.* 2002)

a *Gal4* lines from D.D.-B. lab were generated by combining *Gal4* drivers obtained from the BDSC with *tub-Gal80^ts^* through standard genetic crosses.

For tissue- and cell type-specific transgene expression, females of genotypes *y w*; Gal4*/UAS-transgene or *y w*; *UAS-transgene/+*; Gal4*/+ (Gal4* represents Gal4 lines used in this study) were raised at room temperature, and 0-to-2-day-old females were switched to 29° for 7 days to induce transgene expression. For all experiments, standard medium was supplemented with wet yeast paste.

### Immunostaining and confocal microscopy

Tissues were dissected in Grace’s insect medium with L-glutamine (Caisson Labs) and fixed in 5.3% formaldehyde (Ted Pella) in Grace’s medium at room temperature. Ovaries were teased apart to separate ovarioles and fixed for 13 min; brains and carcasses were fixed for 20 min; thoraces were fixed for 30 min; and guts with attached Malpighian tubules were fixed for one hour. Samples were rinsed three times and washed three times for 15 min in PBSTx (PBS; 10 mM NaH_2_PO_4_/NaHPO_4_, 175 mM NaCl, pH 7.4, 0.1% Triton X-100), and subsequently incubated for three hours at room temperature in blocking solution consisting of 5% normal goat serum (NGS, MP Biomedicals) and 5% bovine serum albumin (BSA, Sigma-Aldrich) in PBSTx. Samples were incubated at 4° overnight in the following primary antibodies diluted in blocking solution: rabbit anti-GFP (Torrey Pines Biolabs Inc, 1:2500); chicken anti-GFP (Abcam, 1:1000); and mouse anti-β-Galactosidase (Promega, 1:500). Samples were rinsed three times and washed three times for 15 min in PBSTx before incubation for two hours at room temperature in 1:400 Alexa Fluor 488-conjugated goat species-specific secondary antibodies (ThermoFisher Scientific). Samples were rinsed, washed, and mounted in Vectashield with 1.5 μg/mL 4’,6-diamidino-2-phenylindole (DAPI) (Vector Laboratories). Images were acquired with a Zeiss LSM700 confocal microscope.

### Data availability

*Drosophila* strains are available upon request. The authors affirm that all data necessary for confirming the conclusions of the article are present within the article, figures, and tables.

## Results And Discussion

### Different hh-Gal4 “niche” drivers have distinct patterns of expression in adult females

**Figure 1:**
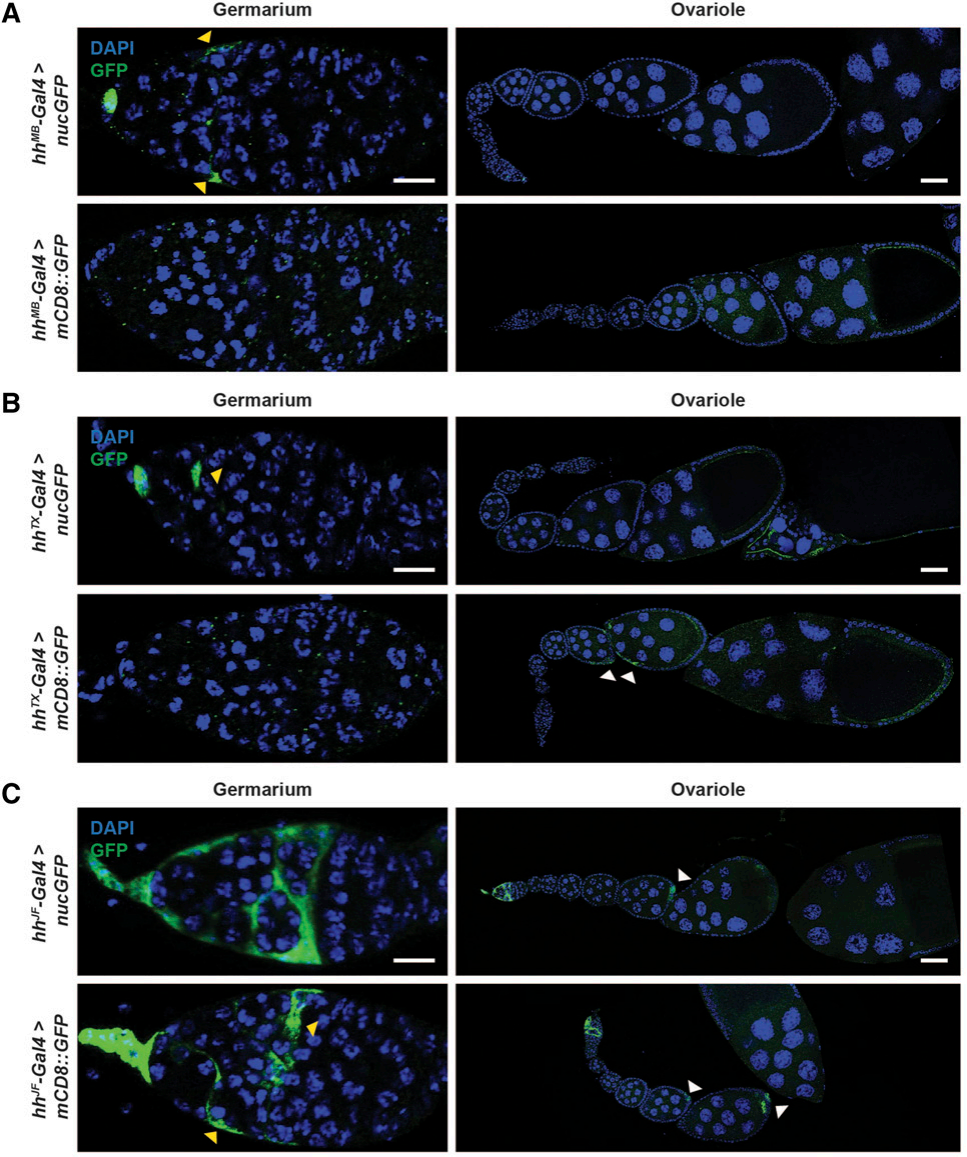
Expression patterns of *hh-Gal4* lines in the adult female ovary. Expression of *UAS-nucGFP* or *UAS-mCD8*::*GFP* induced by “niche” drivers *hh*^*MB*^*-Gal4* (A), *hh*^*TX*^*-Gal4* (B), and *hh*^*JF*^*-Gal4* (C). GFP (green); DAPI (blue), nuclei. Scale bars: 10 µm (germarium); 50 µm (ovariole). Arrowheads point to GFP expression in escort cells (yellow) or later follicle cells (white).

**Figure 2:**
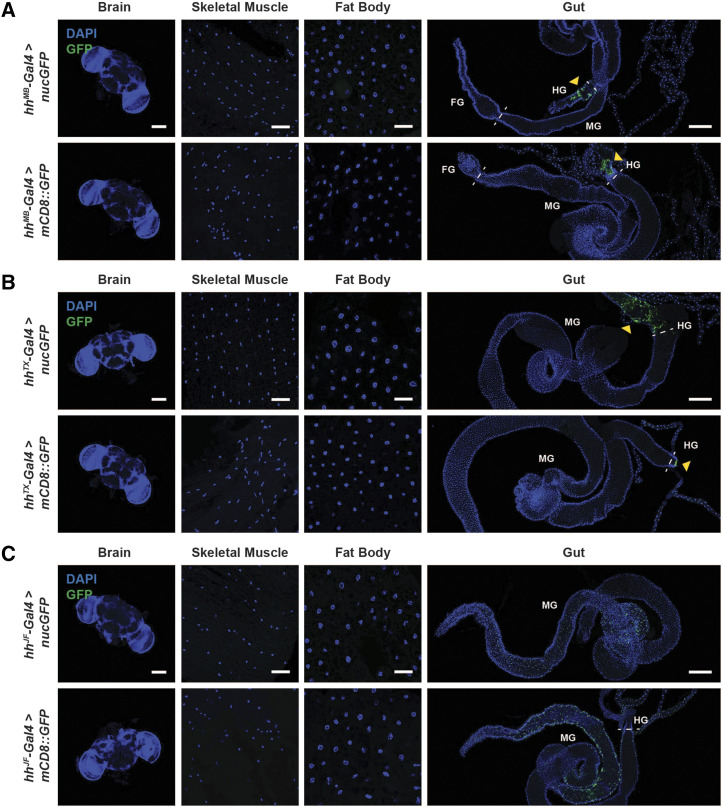
Expression patterns of *hh-Gal4* lines in additional adult female tissues. Expression of *UAS-nucGFP* or *UAS-mCD8*::*GFP* induced by “niche” drivers *hh*^*MB*^*-Gal4* (A), *hh*^*TX*^*-Gal4* (B), and *hh*^*JF*^*-Gal4* (C). GFP (green); DAPI (blue), nuclei. Scale bars: 100 µm (brain); 25 µm (skeletal muscle); 25 µm (fat body); 250 µm (gut). Dashed lines separate sections of the gut. Foregut (FG); midgut (MG); hindgut (HG). Yellow arrowheads point to GFP expression in the hindgut.

*Gal4* drivers expressed in subsets of cells in the adult ovary are routinely used for the study of oogenesis (Hudson and Cooley 2014). To determine the degree of cell type/tissue specificity of commonly used ovary *Gal4* drivers (Table 2), we carefully examined their expression patterns in adult female tissues using *UAS-nucGFP* or *UAS-mCD8*::*GFP* reporters. (Please note that the expression patterns we report throughout this study were consistently observed with 100% penetrance in the analyzed samples.) We first looked at the expression pattern of terminal filament and cap cell drivers known as *hh-Gal4* that were obtained from three independent sources. The first *hh-Gal4* line (an enhancer trap *Gal4* line obtained from Michael Buszczak and referred hereafter as *hh-Gal4^MB^*) (Tanimoto *et al.* 2000; Eliazer *et al.* 2011) drove expression of *UAS-nucGFP* in the cap cells as previously reported (Figure 1A) (Eliazer *et al.* 2011) but was also expressed in some escort cells [which are somatic cells that envelop and support differentiating germ cells in the anterior portion of germarium prior to the envelopment of 16-cell germline cysts by follicle cells (Margolis and Spradling 1995)] (Figure 1A, yellow arrowheads) and in the hindgut (Figure 2A, yellow arrowhead). Surprisingly, *hh-Gal4^MB^* failed to drive expression of *UAS-mCD8*::*GFP* in cap cells or escort cells (Figure 1A); however, *UAS-mCD8*::*GFP*, like *UAS-nucGFP*, was also expressed in the hindgut (Figure 2A). The second *hh-Gal4* tested (obtained from Ting Xie and referred hereafter as *hh-Gal4^TX^*) (Pan *et al.* 2007) drove expression of *UAS-nucGFP* in cap cells and a subset of escort cells (Figure 1B, yellow arrowhead) and in the hindgut (Figure 2B, yellow arrowhead). (Please note that we were unable to find any information about how *hh-Gal4^TX^* was generated.) Like *hh-Gal4^MB^*, however, *hh-Gal4^TX^* did not drive expression of *UAS-mCD8*::*GFP* in the germarium (Figure 1B), but *UAS-mCD8*::*GFP* expression was observed in later stage follicle cells (Figure 1B, white arrowheads) and in some cells in the hindgut (Figure 2B, yellow arrowhead). Lastly, we examined the expression pattern of the Janelia Farm *hh-Gal4* driver (referred hereafter as *hh-Gal4^JF^*), which was generated by subcloning of the *hh* regulatory region upstream of Gal4 and site-specific transgene insertion (Jenett *et al.* 2012). *UAS-nucGFP* driven by *hh-Gal4^JF^* showed robust expression in the terminal filament, cap cells, and escort cells (Figure 1C). *UAS-mCD8*::*GFP* driven by *hh-Gal4^JF^*, however, was much more strongly expressed in the terminal filament and cap cells than in escort cells (Figure 1C). Both GFP constructs were expressed in follicle cells (Figure 1C, white arrowheads) and in the midgut (Figure 2C) when driven by *hh-Gal4^JF^*. These results suggest that different lines termed “*hh-Gal4*” have distinct patterns of expression that are also in part dependent on the type and insertion site of the *UAS* reporter transgene. Thus, depending on the *hh-Gal4* driver used, some result interpretations might be confounded by additional expression in other tissues and ovarian cell types, and not all *UAS* transgenes will necessarily be induced in the expected *hh-Gal4* pattern.

**Table 2:** Expression patterns of Gal4 drivers in adult female tissues

Reported tissue specificity	Driver	Brain	Muscle	Fat Body	Gut	Ovary	Reference
**Ovary**	*bab1-Gal4*	+	—	—	+	+	This study
	*hh-Gal4^MB^*	—	—	—	+	+	This study
	*hh-Gal4^TX^*	—	—	—	+	+	This study
	*hh-Gal4^JF^*	—	—	—	+	+	This study
	*ptc-Gal4*	+	—	—	+	+	This study
	*c587-Gal4*	+	—	+	—	+	This study
	*tj-Gal4*	+	—	+	—	+	This study
**Gut and Malpighian tubules**	*mex1-Gal4*	—	—	—	+	—	This study
	*NP3084-Gal4*	+	—	—	+	—	This study
	*esg-Gal4*	+	—	—	+	—	This study
	*dl-Gal4*	+	—	—	+	+	This study
	*Su(H)GBE-Gal4*	+	—	—	+	+	This study
	*myo31D-Gal4*	+	—	—	+	—	(Weaver and Drummond-Barbosa 2019)
	*c42-Gal4*	+	—	—	+	—	This study
	*Uro-Gal4*	—	+	—	—	—	This study
**Muscle and brain**	*MHC-Gal4*	—	+	—	—	—	(Weaver and Drummond-Barbosa 2019)
	*mef2-Gal4*	+	+	—	+*^a^*	—	This study
	*nSyb.P-Gal4*	+	—	—	+	—	This study
	*nSyb.S-Gal4*	+	—	—	—	—	(Weaver and Drummond-Barbosa 2019)
	*repo-Gal4*	+	—	—	+	—	This study
	*ChAT-Gal4*	+	—	—	—	—	This study
**Sensory neurons**	*pebbled-Gal4*	+	—	—	+	+	This study
	*Gr5a-Gal4*	+	—	—	—	—	This study
	*Gr66a-Gal4*	+	—	—	—	—	This study
	*Ir8a-Gal4*	+	—	—	—	—	This study
	*Ir25a-Gal4*	+	—	—	—	—	This study
	*Or83b-Gal4*	+	—	—	—	—	This study
	*ppk-Gal4*	+	—	—	—	—	This study
**Fat body**	*adh-Gal4*	+	n.d.*^b^*	+	+	+	(Armstrong *et al.* 2014)
	*cg-Gal4*	+	n.d.	+	—	+	(Armstrong *et al.* 2014)
	*FB-Gal4*	—	n.d.	+	+	—	(Armstrong *et al.* 2014)
	*3.1Lsp2-Gal4*	—	n.d.	+	—	—	(Armstrong *et al.* 2014)
	*r4-Gal4*	+	n.d.	+	+	+	(Armstrong *et al.* 2014)
	*ppl-Gal4*	—	n.d.	+	+	—	(Armstrong *et al.* 2014)
	*PromE800-Gal4*	—	—	+	—	—	(Weaver and Drummond-Barbosa 2019)

a Expression in visceral muscle surrounding gut.

b n.d., not determined.

### Ovary Gal4 drivers are expressed in additional tissues in adult females

**Figure 3:**
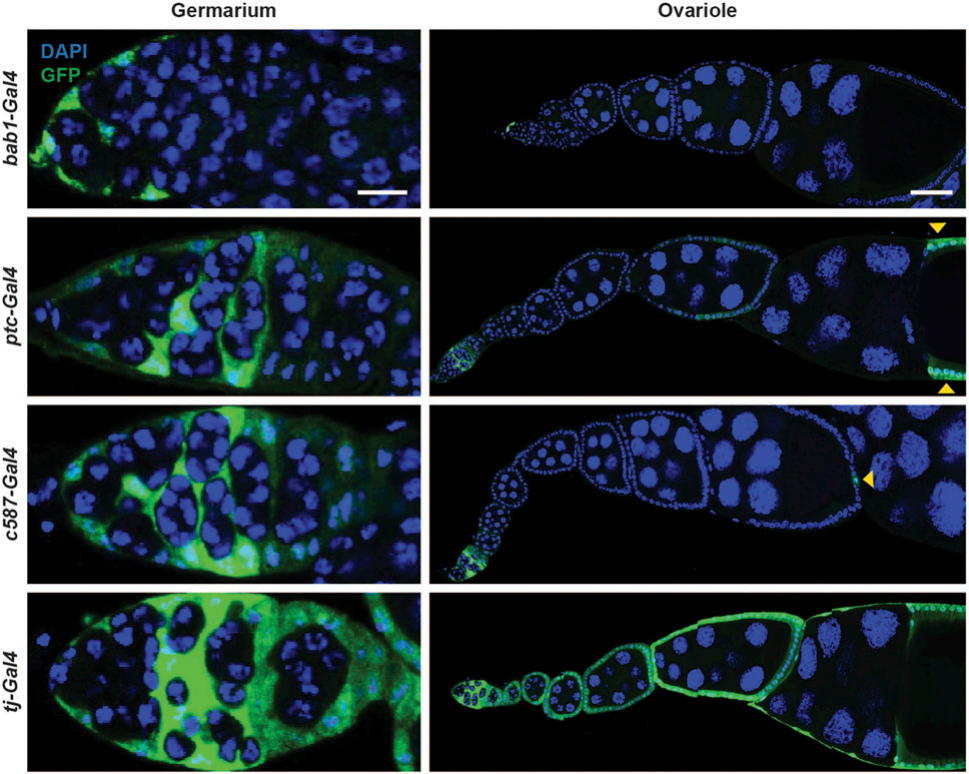
Expression patterns of commonly used ovary *Gal4* drivers in adult female ovaries. Expression of *UAS-nucGFP* induced by the cap cell and escort cell driver *bab1-Gal4*, escort cell driver *ptc-Gal4*, escort cell driver *c587-Gal4*, or follicle cell driver *tj-Gal4*. GFP (green); DAPI (blue), nuclei. Scale bars: 10 µm (germarium); 50 µm (ovariole). Yellow arrowheads point to GFP expression driven by *ptc-Gal4* or *c587-Gal4* in follicle cells.

**Figure 4:**
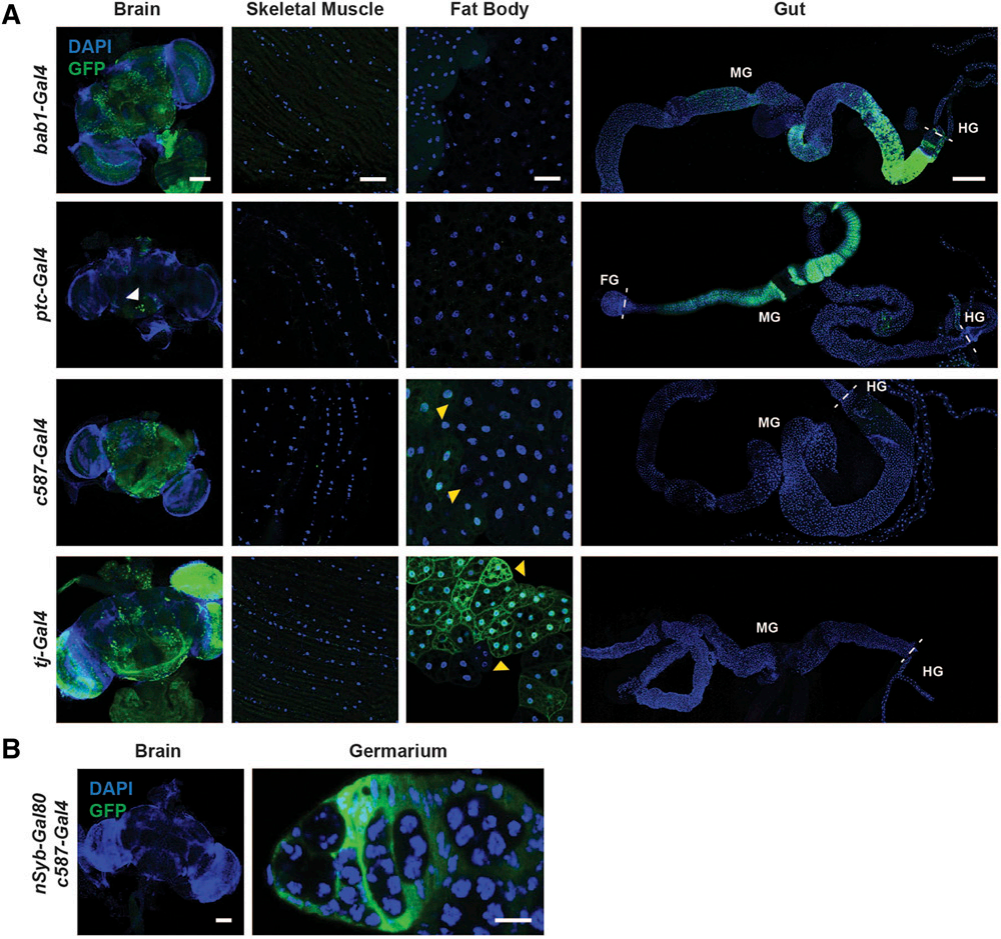
Ovary *Gal4* driver expression patterns in additional adult female tissues. Expression of *UAS-nucGFP* induced by the cap cell and escort cell driver *bab1-Gal4*, escort cell driver *ptc-Gal4*, escort cell driver *c587-Gal4*, or follicle cell driver *tj-Gal4*. GFP (green); DAPI (blue), nuclei. Scale bars: 100 µm (brain); 25 µm (skeletal muscle); 25 µm (fat body); 250 µm (gut). Arrowheads point to some GFP expressing brain cells (white) and adipocytes (yellow). Dashed lines separate sections of the gut. Foregut (FG); midgut (MG); hindgut (HG). (B) Expression of *UAS-nucGFP* induced by *c587-Gal4* in combination with *nSyb-Gal80* showing lack of GFP expression in the brain. GFP (green); DAPI (blue), nuclei. Scale bars: 100 µm (brain), 10 µm (germarium).

In addition to *hh-Gal4*, other *Gal4* drivers are used for specific expression in other cell types found in the adult ovary. Of the drivers we tested, almost all showed expression either outside of the ovary or in an additional unreported ovarian cell type (Figures 3 and 4, Table 2). For example, the cap cell and escort cell driver *bab1-Gal4* (also known as *bab^Agal4-5^*) drove robust expression of *UAS-nucGFP* in the cap cells and escort cells (Figure 3) as reported (Cabrera *et al.* 2002), but also showed strong GFP expression in the brain and midgut (Figure 4A). Although not tested in our study, an additional *bab1-Gal4* line (*bab^Pgal4-2^*) (Cabrera *et al.* 2002) has also been generated and should be carefully characterized in future studies. The escort cell driver *ptc-Gal4* (Forbes *et al.* 1996) induced GFP in ovarian escort cells as previously reported (Figure 3); however, this driver also showed expression in late stage follicle cells (Figure 3, yellow arrowheads), and in some brain cells (Figure 4A, white arrowhead) and the gut (Figure 4A). The escort cell driver *c587-Gal4* (Zhu and Xie 2003; Hsu and Drummond-Barbosa 2009) showed GFP expression in the reported ovarian cell types (Figures 3 and 4B), but showed additional expression in the brain and fat body (Figure 4A), and occasional late stage follicle cells (Figure 3, yellow arrowhead). Finally, the follicle cell driver *tj-Gal4* showed GFP expression in the brain and fat body (Figure 4A) in addition to its reported expression in ovarian follicle cells (Figure 3). These results indicate that commonly used ovary drivers have additional sites of expression in multiple tissues in adult females. To determine whether an effect in the ovary is indeed cell type specific, it will be important to rule out potential roles of additional tissues in which these drivers are expressed.

### Gut, muscle, and Malpighian tubule drivers are expressed in multiple tissues in adult females

**Figure 5:**
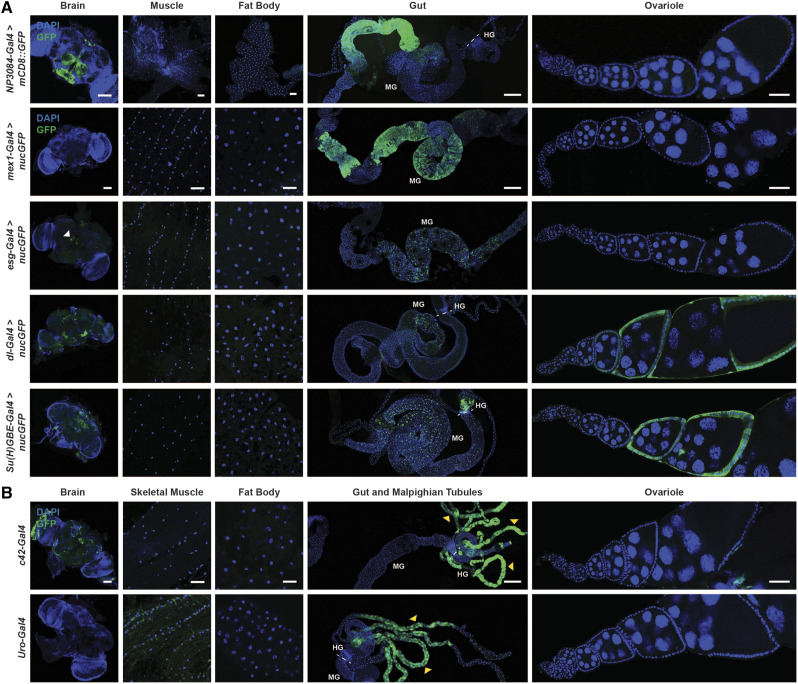
Midgut and Malpighian tubule *Gal4* driver expression patterns in adult *Drosophila* females. (A) Expression of *UAS-mCD8*::*GFP* induced by the midgut driver *NP3084-Gal4*. GFP (green); DAPI (blue), nuclei. Scale bars: 100 µm (brain); 50 µm (skeletal muscle); 50 µm (fat body); 250 µm (gut); 50 µm (ovariole). *UAS-nucGFP* induced by enterocyte driver *mex1-Gal4*, ISC/enteroblast driver *esg-Gal4*, ISC driver *dl-Gal4*, or enteroblast driver *Su(H)GBE-Gal4*. GFP (green); DAPI (blue), nuclei. Scale bars: 100 µm (brain); 25 µm (skeletal muscle); 25 µm (fat body); 250 µm (gut); 50 µm (ovariole). All rows (except for top row) are shown at the same magnification for corresponding tissues. White arrowhead indicates some GFP expressing cells in the brain. (B) Malpighian tubule drivers *c42-Gal4* and *Uro-Gal4* expressing *UAS-nucGFP*. GFP (green); DAPI (blue), nuclei. Scale bars: 100 µm (brain); 25 µm (skeletal muscle); 25 µm (fat body); 250 µm (gut); 50 µm (ovariole). Yellow arrowheads indicate Malpighian tubules. Dashed lines separate sections of the gut. Midgut (MG); hindgut (HG).

We previously confirmed that in adult females the *myo31DF^NP0001^-Gal4* driver (Regan *et al.* 2016) is largely specific for the visceral muscle surrounding the midgut, and showed additional slight expression in the brain (Weaver and Drummond-Barbosa 2019) (Table 2). In addition, *NP3084-Gal4* (Hayashi *et al.* 2002) drove expression of *UAS-mCD8*::*GFP* in the gut as reported (Nehme *et al.* 2007), but also drove expression in the brain (Figure 5A). By contrast, expression of *UAS-nucGFP* under control of the enterocyte driver *mex1-Gal4* (Phillips and Thomas 2006) was restricted to the adult female gut with no GFP expression observed in other tissues (Figure 5A, Table 2). The commonly used ISC/enteroblast driver *esg-Gal4* (Micchelli and Perrimon 2006) showed low levels of GFP in a few cells in the brain in addition to its expression in ISCs and enteroblasts (Figure 5A, Table 2), while both the ISC driver *dl-Gal4* (Zeng *et al.* 2010) and the enteroblast driver *Su(H)GBE-Gal4* (Zeng *et al.* 2010) showed expression in the brain and in follicle cells in the ovary in addition to their reported expression in the midgut (Figure 5A, Table 2). Although often overlooked, these additional sites of Gal4 expression are not surprising, given the known expression pattern/function of the genes whose regulatory regions control these *Gal4* transgenes (Vässin *et al.* 1987; Schweisguth and Posakony 1992; Ashraf *et al.* 1999). For example, Dl was previously shown to be expressed in the follicle cells and the germline throughout oogenesis and is required for fertility (Ruohola *et al.* 1991). Experiments using these midgut cell type drivers for genetic manipulation of adult females should ideally include additional controls to rule out effects of gene manipulation in the brain or follicle cells. Alternatively, these drivers could be combined with tissue-specific Gal80 expression for suppression of Gal4 activity in the additional cell types that are not of interest to avoid confounding effects.

**Figure 6:**
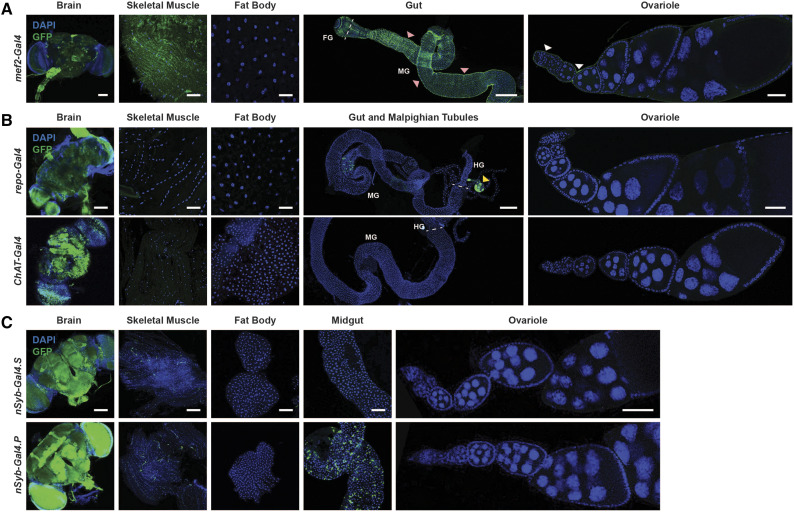
Expression patterns of additional muscle and brain Gal4 drivers in adult females. (A) Expression of *UAS-nucGFP* induced by the muscle driver *mef2-Gal4*. The GFP expression observed in the gut of the *mef2-Gal4* driver represents the visceral muscle. Arrowheads indicate visceral muscle (pink) and ovariole muscle sheath (white). (B) Expression of *UAS-nucGFP* induced by the glial cell driver *repo-Gal4* and of *UAS-mCD8*::*GFP* induced by the cholinergic neuron driver *ChAT-Gal4*. GFP (green); DAPI (blue), nuclei. Scale bars, 100 µm (brain); 25 µm (skeletal muscle); 25 µm (fat body); 250 µm (gut); 50 µm (ovariole). Arrowheads indicate Malpighian tubules (yellow). (C) Expression of *UAS-mCD8*::*GFP* induced by the neuron drivers *nSyb-Gal4.S* and *nSybGal4.P*. GFP (green); DAPI (blue), nuclei. Scale bars, 100 µm (brain, skeletal muscle, fat body, and midgut); 50 µm (ovariole). Dashed lines separate sections of the gut. Foregut (FG); midgut (MG); hindgut (HG).

We also examined the expression patterns of two Malpighian tubule drivers and an additional muscle driver (Figure 5B, Figure 6A). The Malpighian tubule driver *c42-Gal4* (Rosay *et al.* 1997) showed high nucGFP levels in both the Malpighian tubules and in parts of the brain, whereas *Uro-Gal4* (Terhzaz *et al.* 2010) showed low expression of GFP in muscles in addition to its strong expression in Malpighian tubules (Figure 5B, Table 2). We previously showed that *MHC-Gal4* (Schuster *et al.* 1996) is specific for adult female skeletal muscle without expression in additional tissues (Weaver and Drummond-Barbosa 2019) (Table 2). Conversely, analysis of the commonly used *mef2-Gal4* muscle driver (Ranganayakulu *et al.* 1998) shows robust expression in the brain in addition to skeletal and visceral (around the gut) muscles (Figure 6A, Table 2). These results suggest that when using drivers for Malpighian tubule-specific manipulation or *mef2-Gal4* for muscle-specific experiments, the expression in additional tissues with these drivers should be either blocked with Gal80 or functionally evaluated using other drivers.

### Commonly used sensory neuron drivers are highly specific in adult females

**Figure 7:**
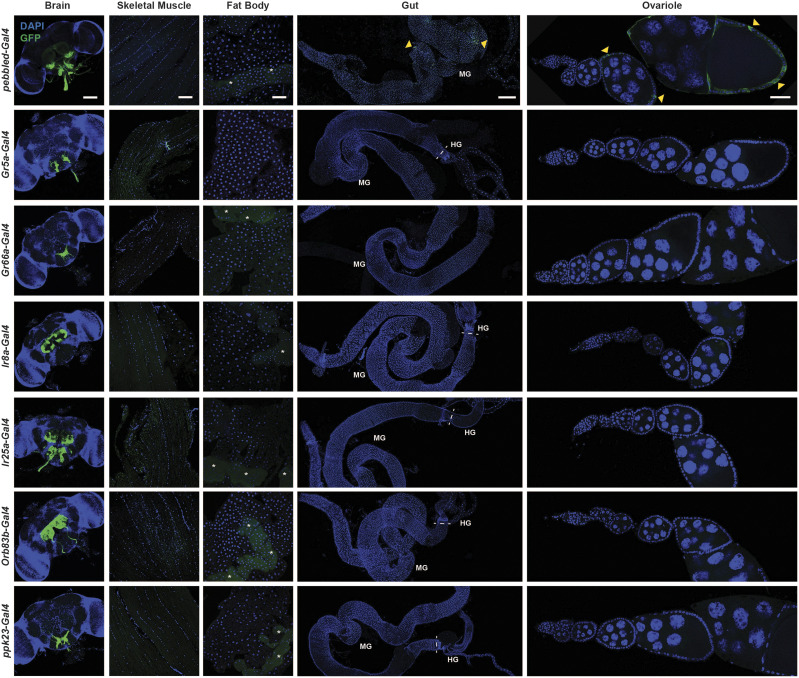
Sensory neuron Gal4 driver expression in adult females. Expression of *UAS-mCD8*::*GFP* induced by sensory neuron drivers. GFP (green); DAPI (blue), nuclei. Scale bars, 100 µm (brain); 25 µm (skeletal muscle); 25 µm (fat body); 250 µm (gut); 50 µm (ovariole). The faint green fluorescence observed in the fat body images results from oenocyte autofluorescence (asterisks). Dashed lines separate sections of the gut. Midgut (MG); hindgut (HG).

We previously confirmed that in adult females the pan-neuronal driver *nSyb-Gal4.S* (Pauli *et al.* 2008) is exclusively expressed in the brain (Weaver and Drummond-Barbosa 2019) (Table 2; also see Figure 6C). Similarly, the cholinergic neuron driver *ChAT-Gal4* (Salvaterra and Kitamoto 2001) drives *UAS-mCD8*::*GFP* expression only in the brain (Figure 6B, Table 2). By contrast, the glial cell driver *repo-Gal4* (Sepp *et al.* 2001) exhibits some nucGFP expression in the Malpighian tubules in addition to its reported expression in the brain (Figure 6B). In addition to these more broadly expressed brain drivers, we also analyzed multiple sensory neuron drivers using the *UAS-mCD8*::*GFP* reporter for their level of specificity (Figure 7). Most of the sensory neuron drivers tested showed highly specific expression in the brain, without additional expression in other tissues. These results are perhaps not surprising given that all sensory neuron drivers we tested are driven by small, gene-specific regulatory regions, ranging in size from 215 bp (*Ir8a-Gal4*) (Abuin *et al.* 2011) to 7.4 kb (*ChAT-Gal4*) (Salvaterra and Kitamoto 2001). The specialized functions of these genes, most of which encode olfactory and gustatory receptors (Chen and Dahanukar 2020), may also contribute to their specificity of expression. One exception was *pebbled-Gal4*, which showed additional expression in late ovarian follicle cells and in some cells in the gut (Figure 7). However, the expression pattern of *pebbled-Gal4* is unsurprising given the known roles of *pebbled* in promoting the mitotic-to-endocycle switch in follicle cells and follicle cell differentiation (Sun and Deng 2007), and its known expression in the gut (Celniker *et al.* 2009). Collectively, these results suggest that neuronal drivers in general are more likely to be specifically expressed in neurons, perhaps in part due to the highly specialized nature of these cells. However, additional neuronal drivers still need to be tested to ensure that expression patterns are specific to their neuronal cell population of interest.

## Conclusions and other considerations for future studies

Many adult tissues produce systemic factors, including peptide hormones, lipids and other types of molecules to modulate the function of multiple tissues within an organism (Droujinine and Perrimon 2016; Castillo‐Armengol *et al.* 2019; Drummond-Barbosa 2019). Dissecting the complexity of inter-organ signaling networks requires reliable tools for tissue-specific genetic manipulation. This study highlights that many *Gal4* drivers commonly used for tissue-specific manipulation of gene function have previously unreported additional sites of expression in adult *Drosophila* females. These findings are of concern to *Drosophila* researchers because expression of Gal4 drivers in multiple tissues can confound the interpretation of results aimed at evaluating tissue-specific effects of gene manipulation on a given tissue/biological process.

To ensure that manipulations are indeed tissue-specific, it is crucial to thoroughly test drivers and document their expression patterns broadly across *Drosophila* tissues according to the specifics of each study. For example, Gal4 expression patterns should be analyzed in specific developmental stages of interest (*e.g.*, larvae *vs.* adults), in males *vs.* females, under the specific dietary conditions of the experiment, and in response to any additional physiological conditions considered during the course of a study. Simply put, it would not be wise to assume that the published expression pattern of any given *Gal4* driver will remain the same under the specific experimental conditions of a particular study.

While many of the drivers tested are expressed in previously unreported tissues, there are known ways to eliminate expression in secondary tissues by using the Gal4 inhibitor Gal80 (Stoleru *et al.* 2005; Xie *et al.* 2018). For example, *nSyb-Gal80* is routinely used in combination with *Gal4* drivers to inhibit Gal4 specifically in neurons and allow *UAS-GFP* (or other transgene) expression only in the remaining tissue of interest (Rubinstein *et al.* 2010). In accordance, we successfully combined *c587-Gal4* with *nSyb-Gal80* to eliminate the neuronal expression observed in the brain with the *c587-Gal4* driver alone, without affecting expression in the ovary (Figure 4B). Analogously, *Su(H)GBE-Gal80* is commonly used to inhibit Gal4 in enteroblasts and thus restrict expression of *esg-Gal4* to only ISCs (Wang *et al.* 2014). Evidently, any *Gal4* driver could potentially be combined with cell type/tissue-specific *Gal80* transgenes to limit Gal4 activity to desired target tissues. However, if a *Gal80* transgene is not available for a specific tissue, effects from secondary tissues that express the Gal4 targeting the cell type/tissue of interest could be ruled out by using a separate *Gal4* driver specific for that secondary cell type/tissue.

Alternatively, a combinatorial approach commonly used in the *Drosophila* neuroscience field to generate neuronal type-specific drivers can also be used more broadly to generate cell type/tissue-specific drivers. In this approach, the Gal4 transcription factor is subdivided into its DNA-binding domain (DBD) and its activating domain (AD), and only cells which express both of these components are able to produce a functional Gal4 to induce UAS transgene expression (Xie *et al.* 2018). By expressing DBD and AD under control of separate enhancers/promoters, it is possible to achieve expression in only the tissues where the expression pattern induced by the two regulatory regions overlap. For example, a truly Malpighian tubule-specific driver could be generated by combining c42-DBD with Uro-AD, since the only tissues in which these two promoters overlap are the Malpighian tubules (Figure 5B). These DBD and AD lines can be generated from existing Gal4 lines using Homology Assisted CRISPR Knock-in (HACK) (Lin and Potter 2016). HACK uses CRISPR-Cas9 technology to induce double-stranded breaks in *Gal4* transgenes, which is repaired by a transgenic construct containing *Gal4* homologous sequences flanking a cassette (*e.g.*, *DBD* or *AD*) to replace the *Gal4* transgene. This method has been successfully used to generate *TH-AD*, *TH-DBD*, and *TH-Gal80* transgenic lines (Xie *et al.* 2018). Although more labor intensive, having highly specific tools or strategies to rule out effects from other tissues is highly advantageous as we strive for an accurate understanding of complex functional inter-organ relationships.

In addition to the promoter sequence directly upstream of *Gal4*, the site of the insertion of the *Gal4* transgenes can also affect tissue-specific expression. For example, we previously reported that the *nSyb-Gal4.S* line (Pauli *et al.* 2008) is expressed only in neurons and in no other tissues (Weaver and Drummond-Barbosa 2019); however, a different *nSyb-Gal4* line using the same regulatory sequence but generated by site specific insertion (*nSyb-Gal4.P*) (Riabinina *et al.* 2015) has additional expression in the gut (Figure 6C). Therefore, for *Gal4* transgenes inserted in different sites along the genome (even under the same regulatory region), it is important to validate each line to ensure that there are no additional sites of expression due to the insertion site.

**Figure 8:**
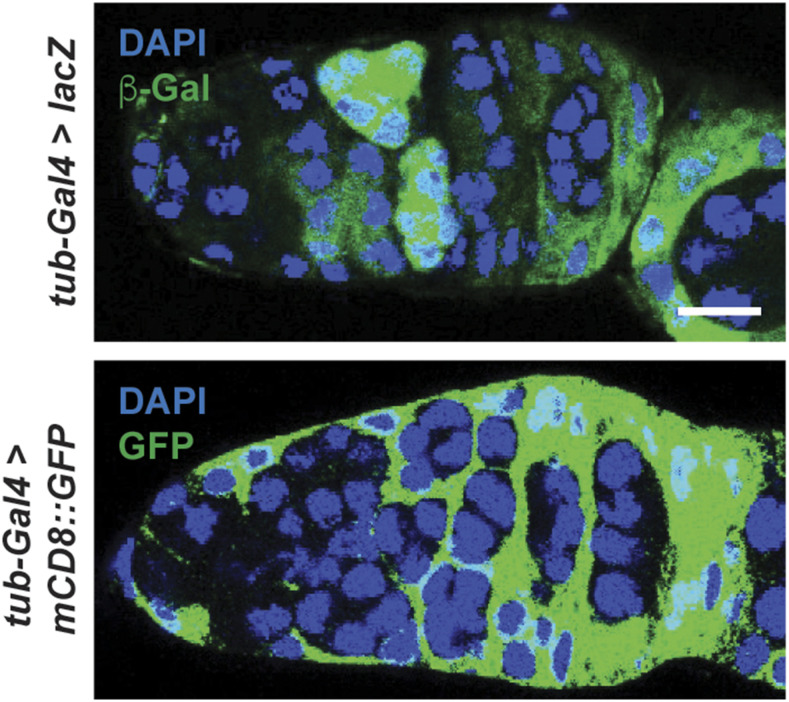
Expression of *UASp* *vs.* *UASt* reporters differs in response to the ubiquitous *tub-Gal4* driver. Expression of *UASp-lacZ* and *UAS-mCD8*::*GFP* induced by *tub-Gal4*, illustrating how reporter type can affect recognized Gal4 pattern. β-gal (green); GFP (green); DAPI (blue), nuclei. Scale bar, 10 µm.

The *UAS* responder transgene should also be taken into consideration for tissue-specific manipulations. For example, it is well known that *UASt* transgenes (referred throughout this study as simply *UAS*) (Brand and Perrimon 1993) are strongly expressed in somatic cells but show limited, if any, expression in the female germline, whereas the *UASp* (Rørth 1998) and *UASz* (Deluca and Spradling 2018) transgenes have been optimized for expression in the female germline. When validating the Gal4 expression pattern of a driver, it would be advisable to use a reporter transgene built using the same *UAS* vector type as the *UAS* transgenes intended for experimental manipulations. To illustrate this point, we crossed the ubiquitous *tub-Gal4* driver to *UASp-lacZ* or *UAS-mCD8*::*GFP*, which resulted in reporter expression predominantly in germ cells or exclusively in somatic cells in the germarium, respectively (Figure 8). Beyond that, we also documented that even distinct reporter lines built using the same *UAS* vector can also show differences in expression under control of the same *Gal4* driver. For example, expression of *UAS-mCD8*::*GFP* with the *hh-Gal4^JF^* driver is most strongly expressed in the terminal filament and cap cells of the germarium, with weaker GFP signal in the escort cells (Figure 1). However, uniform expression across all three ovarian cell types was observed using *UAS-nucGFP* under control of *hh-Gal4^JF^* (Figure 1). Furthermore, both *hh-Gal4^MB^* and *hh-Gal4^TX^* were able to drive expression of *UAS-nucGFP*, but not of *UAS-mCD8*::*GFP*, in cap cells and escort cells (Figure 1), suggesting that these differences are possibly due to reporter insertion site. Indeed, differences in variegation occur due to differences in chromatin accessibility, which have been shown to alter Gal4 expression patterns (Tulin *et al.* 2002). Therefore, it would be ideal to confirm Gal4 expression patterns with reporters not only made using the same vector, but that also have the same insertion sites.

Finally, as mentioned above, changes in the external environment or physiology (such as diet, age, infection, temperature, or other stressors) can potentially alter the expression strength or pattern of a driver. For example, expression of the *3.1Lsp2-Gal4* driver (Lazareva *et al.* 2007) on a yeast-free diet is dramatically reduced compared to that on a yeast-rich diet (Armstrong *et al.* 2014). In addition, although the *UAS/Gal4* system itself shows temperature dependence even in the absence of *Gal80^ts^* (Brand *et al.* 1994), it is also possible that the regulatory regions driving Gal4 might respond in different ways to more subtle changes in temperature than those that activate heat-shock-inducible-Gal4, for instance (Brand *et al.* 1994). In addition to considering that common manipulations such as changes in diet can alter the expression of *Gal4* drivers used for genetic manipulations, one should also evaluate the potential effects of the genetic manipulations themselves on driver expression over the course of the experiment.
